# Estimating autozygosity from high-throughput information: effects of SNP density and genotyping errors

**DOI:** 10.1186/1297-9686-45-42

**Published:** 2013-10-29

**Authors:** Maja Ferenčaković, Johann Sölkner, Ino Curik

**Affiliations:** 1 Department of Animal Science, Faculty of Agriculture, University of Zagreb, Svetosimunska 25, 10000 Zagreb, Croatia; 2 Department of Sustainable Agricultural Systems, Division of Livestock Sciences, University of Natural Resources and Life Sciences Vienna, Gregor Mendel Str. 33, A-1180 Vienna, Austria

## Abstract

**Background:**

Runs of homozygosity are long, uninterrupted stretches of homozygous genotypes that enable reliable estimation of levels of inbreeding (i.e., autozygosity) based on high-throughput, chip-based single nucleotide polymorphism (SNP) genotypes. While the theoretical definition of runs of homozygosity is straightforward, their empirical identification depends on the type of SNP chip used to obtain the data and on a number of factors, including the number of heterozygous calls allowed to account for genotyping errors. We analyzed how SNP chip density and genotyping errors affect estimates of autozygosity based on runs of homozygosity in three cattle populations, using genotype data from an SNP chip with 777 972 SNPs and a 50 k chip.

**Results:**

Data from the 50 k chip led to overestimation of the number of runs of homozygosity that are shorter than 4 Mb, since the analysis could not identify heterozygous SNPs that were present on the denser chip. Conversely, data from the denser chip led to underestimation of the number of runs of homozygosity that were longer than 8 Mb, unless the presence of a small number of heterozygous SNP genotypes was allowed within a run of homozygosity.

**Conclusions:**

We have shown that SNP chip density and genotyping errors introduce patterns of bias in the estimation of autozygosity based on runs of homozygosity. SNP chips with 50 000 to 60 000 markers are frequently available for livestock species and their information leads to a conservative prediction of autozygosity from runs of homozygosity longer than 4 Mb. Not allowing heterozygous SNP genotypes to be present in a homozygosity run, as has been advocated for human populations, is not adequate for livestock populations because they have much higher levels of autozygosity and therefore longer runs of homozygosity. When allowing a small number of heterozygous calls, current software does not differentiate between situations where these calls are adjacent and therefore indicative of an actual break of the run versus those where they are scattered across the length of the homozygous segment. Simple graphical tests that are used in this paper are a current, yet tedious solution.

## Background

Runs of homozygosity (ROH) are continuous stretches of homozygous genotypes without heterozygosity in the diploid state. Although ROH can arise by different mechanisms [1], the primary cause is believed to be inbreeding [2]. Long ROH are most likely the result of recent inbreeding, where recombination events do not shorten identical haplotypes inherited from the common ancestor. Short ROH, in contrast, suggest more ancient inbreeding. The ability of ROH to reveal information about ancient and recent genetic events makes them useful tools to analyze population history [3], inbreeding levels [4] and effects of inbreeding on complex traits and congenital disorders [5].

While ROH from high-throughput genotyping analyses have been studied extensively in humans, such analyses are rare in cattle and other livestock species [6-10]. The lack of standards for ROH definition and identification may introduce bias in ROH-based estimates of autozygosity. Howrigan et al. [11] found that the numbers and sizes of ROH that are identified in genotyping data can strongly depend on certain parameters and thresholds imposed during sequence analysis. In addition, pruning single nucleotide polymorphisms (SNPs) that show low minor allele frequency (MAF), that deviate from Hardy-Weinberg equilibrium (HWE), or that show high linkage disequilibrium (LD), can affect the results [12,13].

The density of the SNP chip used to generate the data for ROH identification is another factor that strongly affects autozygosity estimates. Purfield et al. [6] compared estimates obtained using the two SNP chips most frequently used in cattle: the Illumina BovineSNP50 Genotyping BeadChip with 54 001 SNPs (50 k) and the Illumina BovineHD Genotyping BeadChip with 777 972 SNPs (HD). They concluded that the 50 k chip is appropriate only for identifying ROH longer than 5 Mb. Indeed, analyses based on lower-density chips can fail to detect heterozygous SNP genotypes that are present in observed ROH.

The frequency of SNP genotyping errors is another factor that can affect ROH-based estimates of autozygosity. Since this frequency usually varies between 0.2% and 1.0% [11,14], it may affect identification of very long ROH that contain numerous SNPs. In fact, any genotyping error, whether homozygote to heterozygote or vice versa, can affect the determination of ROH. A potential solution is to allow a certain number of SNPs to be heterozygous [1], but whether this compromises the reliability of ROH analyses has not been systematically analyzed.

The aim of this study was to analyze the identification of ROH of different length categories and the estimation of genomic inbreeding coefficients based on ROH in three cattle breeds (Brown Swiss, Pinzgauer, Tyrol Grey). Our study focused on the effects of chip density (777 972 versus 54 001 SNPs) and genotyping errors. Results demonstrate, both graphically and statistically, that density of SNP chips affects ROH detection and subsequent estimation of inbreeding levels. The optimal number of heterozygous SNPs allowed during ROH analysis was found to depend on chip density and ROH length.

## Methods

### Genotype data and quality control

The semen samples of the animals included in this study used for DNA extraction and genotyping, were obtained from AI centers through their routine practice in the framework of breeding programs. Therefore, no ethical approval was required for sampling of biological material. DNA samples were obtained from 277 bulls of three breeds: Brown Swiss, 46; Pinzgauer, 118; and Tyrol Grey, 113. Mean pedigree-based inbreeding coefficients (and ranges) were as follows: Brown Swiss, 0.033 (0.009-0.096); Pinzgauer, 0.019 (0–0.088); and Tyrol Grey, 0.022 (0–0.169). The mean complete generation equivalent (see e.g., [15] was highest for Brown Swiss (7.32 generations) and lowest for Pinzgauer (5.32 generations). DNA samples were genotyped using the BovineHD Bead Chip (Illumina Inc., San Diego, CA), which contains 777 972 SNPs; this data set is referred to hereafter as the high-density (HD) panel. For comparison, we extracted and retained SNPs from this panel that were common to both the HD panel and the bovine SNP50 Beadchip v1 (Illumina Inc., San Diego, CA), which contains 54 001 SNPs and which will be referred to in the remainder as the 50 k panel.

Data extraction and quality control were performed separately for each breed. We excluded all SNPs that had not been assigned to a chromosome or that had been assigned to chromosomes X or Y or to the mitochondrial genome. We also excluded SNPs for which more than 10% of genotypes were missing and SNPs with an Illumina GenCall score ≤ 0.7 or an Illumina GenTrain score ≤ 0.4. Two Tyrol Grey bulls were excluded from further analysis because more than 5% of their genotypes were missing. In doing this, our objective was to exclude poorly performing loci and minimize risk of genotyping errors. After quality control, the numbers of SNPs in the HD and 50 k panels were as follows for each breed: Brown Swiss, 615 618 and 38 710; Pinzgauer, 606 120 and 38 198; and Tyrol Grey, 684 172 and 42 997.

Although it is customary in genome-wide association studies and ROH analyses to exclude SNPs with low MAF or high LD with neighboring SNPs or that deviate from HWE, we did not apply such exclusion criteria in our study. Instead we relied on Illumina quality scores (GenCall, GenTrain) to reduce genotyping problems. We also defined the minimum ROH length as 1 Mb to exclude short, common ROH arising from LD [3,6].

### ROH calling options

ROH were identified in every individual using the SNP & Variation Suite (v7.6.8 Win64; Golden Helix, Bozeman, MT, USA http://www.goldenhelix.com). This algorithm is designed to find stretches of consecutive homozygous SNPs; it works continuously across an entire chromosome, examining every possible run that matches the user-specified parameters. We chose this software instead of the PLINK ROH algorithm [16], which uses a sliding window that may introduce artificial runs and fail to identify segments longer than the window.

ROH exceeding the allowed number of heterozygotes or missing SNPs were checked automatically to determine whether they should be removed based on their length, SNP density, and user-specified parameters. ROH were called if 15 or more consecutive homozygous SNPs [17] were present at a density of at least 1 SNP every 100 kb, with gaps of no more than 1000 kb between them. These density and gap thresholds were applied to SNPs in both the HD and 50 k panels to ensure comparability of the results.

Five categories of ROH length (in Mb) were defined: [1,2], (2, 4], (4, 8], (8, 16], and >16. The number of heterozygous SNPs allowed was set to different values for different length categories. First, we called ROH without allowing any heterozygous calls, and we obtained the average numbers of SNPs in each length category (Table 1). We then assumed a genotype error rate of 0.25%, recalculated the numbers of heterozygote calls allowed, and rounded the number of heterozygous SNPs allowed to the nearest whole number. This approach led to the following numbers of heterozygous SNPs allowed for each length category (in Mb) in the HD panel: [1,2], one heterozygeous SNP; (2, 4], two heterozygous SNPs; (4, 8], four heterozygous SNPs; (8, 16], eight heterozygous SNPs; and >16, 16 heterozygous SNPs (Table 2, class C). In the case of the 50 k panel, we allowed one heterozygous SNP for length category >16, and no heterozygous SNPs for the other categories (Table 2, class A).

**Table 1:** Summary statistics for the numbers of SNPs in ROH of different length categories

**Panel**	**Statistic**	**ROH length category (in Mb)**				
		**[1,2]**	**(2, 4]**	**(4, 8]**	**(8, 16]**	**>16**
**50 k panel**	**mean**	21.69	45.13	90.95	178.77	399.39
	**std**	5.68	12.9	23.31	43.17	156.13
	**min**	15.00	21.00	44.00	92.00	210.00
	**max**	49.00	98.00	195.00	354.00	1360.00
**HD panel**	**mean**	291.29	694.25	1432.46	2856.02	6385.9
	**std**	138.56	207.76	361.29	633.38	2377.02
	**min**	15.00	31.00	90.00	1834.00	3617.00
	**max**	808.00	1353.00	2668.00	4825.00	20325.00

Summary statistics were calculated from ROH identified when no heterozygous calls are allowed.

**Table 2:** Definition of classes according to the maximum number of heterozygous SNPs allowed (values in columns) within ROH length categories

**Panel**	**Class**	**ROH length category (in Mb)**				
		**[1, 2]**	**(2, 4]**	**(4 8]**	**(8, 16]**	**>16**
**50 k**	**A**	0	0	0	0	1
	**B**	.	.	.	.	0
**HD**	**C**	1	2	4	8	16
	**D**	0	1	2	4	8
	**E**	.	0	1	2	4
	**F**	.	.	0	1	2
	**G**	.	.	.	0	1
	**H**	.	.	.	.	0

Dots indicate that the value of 0 (no heterozygous allowed) for the given length category was reached in a previous class of the same panel and information is not repeated.

Like the number of heterozygous SNPs, we set the number of missing SNPs allowed to different values for different length categories. First, we determined ROH allowing any number of missing SNPs and then used the results to set limits. This approach led to the following limits for missing SNPs for each ROH length category (in Mb) in the HD and 50 k panels, respectively: [1,2], four or no missing SNPs; (2, 4], eight or no missing SNPs; (4, 8], 16 or one missing SNP; (8, 16], 32 or two missing SNPs; and > 16, 64 or four missing SNPs.

### Calculating inbreeding coefficients from runs of homozygosity (*F*_ROH_)

Statistically *F*_ROH_ is defined as the length of the autosomal genome present in ROH, divided by the overall length of the autosomal genome covered by the SNPs [18]. For each bull, we calculated *F*_ROH>1 Mb_, *F*_ROH>2 Mb_, *F*_ROH>4 Mb_, *F*_ROH>8 Mb_ and *F*_ROH>16 Mb_ based on ROH of different minimum lengths (> 1, > 2, > 4, > 8 or > 16 Mb). *F*_ROH_ was calculated for different minimum ROH lengths because lengths of autozygous segments in a genome are predicted to show an exponential distribution, with a mean length equal to 1/2 g Morgan, where g is the number of generations since the common ancestor (e.g. [11]). If the genome of an individual contains segments as short as 1 Mb, we can conclude that the individual’s autozygosity originated from common ancestors up to 50 generations in the past. Based on the *F*_ROH_ values across all ROH lengths, detected with both 50 k and HD panel, correlations with pedigree inbreeding coefficients were calculated in order to investigate their relationships.

### Identifying significant differences in autozygosity estimates based on the number of heterozygous calls allowed

Mean values of *F*_ROH_ were calculated within classes (scenarios) in which different numbers of heterozygous SNPs were allowed in each ROH length category. Eight classes (A to H) were defined, two (A and B) for the 50 k panel and six (C-H) for the HD panel (Table 2). Numbers of heterozygous SNPs allowed within a class were based on the average numbers of SNPs in a length category and an assumed genotyping error rate of 0.25% for classes A and C. The other classes were formed by successively halving the allowed number of heterozygotes and only considering longer segments (see Table 2).

Mean *F*_ROH_ values obtained when allowing different numbers of heterozygous SNPs were compared within the same length category using paired t-tests. In addition, *F*_ROH_ values were compared between the 50 k and HD panels. The SAS 9.3 [19] procedure TTEST with the PAIRED statement was used to generate p values. The step-down Bonferroni method of Holm [20] using the MULTTEST procedure and the HOLM statement was used to adjust the p values of the 186 comparisons.

## Results and discussion

### Impact of SNP chip density on ROH identification

**Figure 1:**
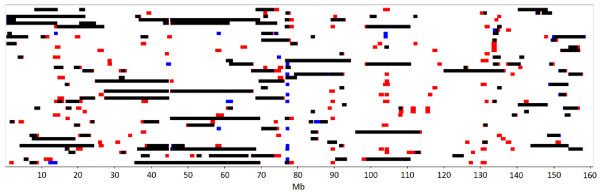
**Overlay of ROH identified on chromosome 1 in Brown Swiss animals.** ROH were identified using 50 k and HD panel data and then overlaid; each row represents one animal, and different colors were used to indicate whether ROH segments were identified using both the 50 k and HD panel (black), only the 50 k panel (red), or only the HD panel (blue).

Across all three cattle breeds, we identified 19 392 ROH segments using the 50 k panel and 14 148 ROH segments using the HD panel (Table 3). For all three breeds, analysis with the 50 k panel identified more ROH > 1 Mb than the HD panel. The two panels gave similar numbers of ROH > 4 Mb. As ROH length increased, the HD panel yielded a higher number of ROH than the 50 k panel (Figure 1). The 50 k panel revealed an abundance of small segments and overestimated the numbers of segments 1–4 Mb long, suggesting that it is not sensitive enough for the precise determination of small segments.

**Table 3:** Summary statistics per breed for the numbers of ROH of different minimum lengths

**Breed**	**ROH length (Mb)**	**Panel**	**Mean**	**std**	**min**	**max**
**Brown Swiss**	**>1**	**50 k HD**	94.76	14.55	66.00	136.00
			82.02	15.48	60.00	150.00
	**>2**	**50 k HD**	47.98	11.66	27.00	81.00
			46.59	9.88	31.00	81.00
	**>4**	**50 k HD**	24.85	6.64	11.00	42.00
			25.93	6.63	13.00	40.00
	**>8**	**50 k HD**	11.50	4.54	3.00	25.00
			12.48	4.66	2.00	23.00
	**>16**	**50 k HD**	3.96	1.89	0.00	8.00
			4.33	2.01	0.00	9.00
**Pinzgauer**	**>1**	**50 k HD**	59.96	9.91	33.00	84.00
			43.26	9.97	19.00	95.00
	**>2**	**50 k HD**	19.44	6.01	5.00	34.00
			19.08	6.66	5.00	46.00
	**>4**	**50 k HD**	8.85	3.93	2.00	20.00
			9.47	4.48	1.00	22.00
	**>8**	**50 k HD**	4.09	2.55	0.00	11.00
			4.41	2.67	0.00	12.00
	**>16**	**50 k HD**	1.36	1.37	0.00	6.00
			1.36	1.39	0.00	6.00
**Tyrol Grey**	**>1**	**50 k HD**	70.86	9.51	52.00	102.00
			44.94	12.14	24.00	100.00
	**>2**	**50 k HD**	21.08	7.94	4.00	55.00
			18.72	7.24	6.00	50.00
	**>4**	**50 k HD**	9.99	5.19	1.00	33.00
			9.60	5.00	1.00	31.00
	**>8**	**50 k HD**	4.43	3.34	0.00	20.00
			4.65	3.29	0.00	21.00
	**>16**	**50 k HD**	1.64	1.90	0.00	12.00
			1.70	1.87	0.00	12.00

The 50 k panel did, however, prove suitable for detecting segments longer than 4 Mb. This finding is consistent with that of Purfield et al. [6], who concluded that the 50 k panel recognizes only segments longer than 5 Mb as well as the HD panel does.

**Figure 2:**
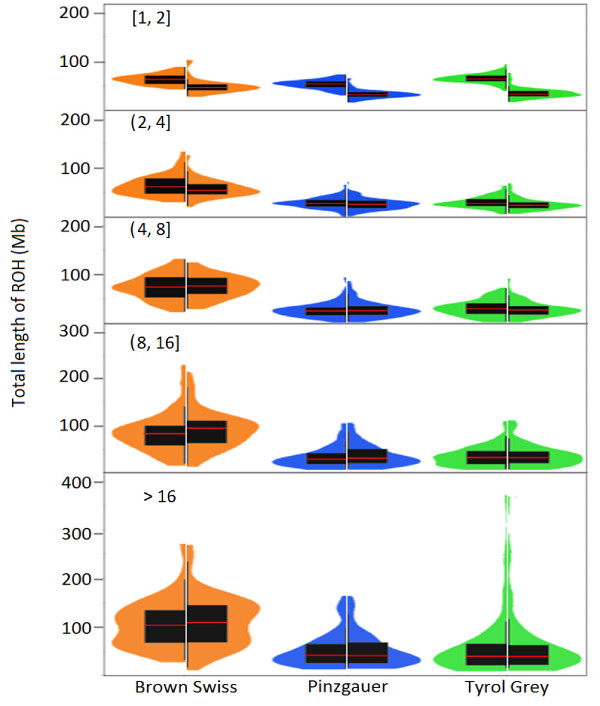
**Overlay of box plots and kernel density plots.** Overlay of box plots and kernel density plots that show the distribution of the total ROH length among all Brown Swiss bulls (orange), Pinzgauer bulls (blue) and Tyrol Grey bulls (green) for five ROH length categories; box plots (black) are shown inside the density plots, and horizontal red lines indicate mean values; the left half of each density and box plot was obtained from the 50 k panel data, while the right half was obtained from the HD panel data.

The 50 k and HD panels gave noticeably different distributions and mean values of ROH length within each length category (Figure 2). Differences were greatest for the [1,2] length category, and then gradually disappeared as ROH length increased. These findings provide further evidence that data from the 50 k panel lead to imprecise determination of short ROH and overestimation of *F*_ROH_.

### Impact of genotyping errors on autozygosity estimates

To our knowledge, a simulation study by Howrigan et al. [11] is the only source of recommendations on the number of heterozygous calls allowed in ROH. They suggested allowing no heterozygous calls. However, since genotyping errors in SNP chip data do occur, it seems more reasonable to allow some heterozygous calls, particularly for ROH > 8 Mb on dense SNP chips. These long segments are much more frequent in cattle populations than in human populations, even for population isolates (e.g. [21]). We determined the numbers of SNPs in ROH of specific lengths and assumed a 0.25% rate of genotyping errors in order to define the number of heterozygous genotypes allowed separately for each ROH length category. Then, we determined mean *F*_ROH_ values for the classes defined in Table 2 for different allowed numbers of heterozygous calls. Paired t-tests were conducted within the eight classes (A-H) within the same length category and within each cattle breed (Table 4). The 50 k and HD panel data gave significantly different mean *F*_ROH>1 Mb_ values in Pinzgauer and Tyrol Grey cattle, and significantly different mean *F*_ROH>4 Mb_ and *F*_ROH>8 Mb_ values in the Brown Swiss and Pinzgauer breeds. For all three breeds, mean *F*_ROH >16 Mb_ based on the 50 k panel differed significantly depending on whether one (class A) or no (class B) heterozygous calls were allowed. These differences had important effects on estimates of inbreeding levels. For each breed, inbreeding levels based on *F*_ROH >16 Mb_ based on the HD panel differed by approximately 1.7-fold, depending on whether 16 or no heterozygous calls were allowed (Table 4). In fact, inbreeding coefficients derived from ROH > 16 Mb with no allowance for heterozygous calls were lower than inbreeding coefficients estimated from pedigrees. These findings suggest that for such long ROH, which can have more than 5000 to 6000 SNPs, some heterozygous calls must be allowed due to the possibility of genotyping errors.

**Table 4:** **Comparison of *F***_**ROH **_**values obtained by allowing different numbers of heterozygous SNPs**

**Breed**	**Source**	**Class**	***F***_**ROH>1**_	***F***_**ROH>2**_	***F***_**ROH>4**_	***F***_**ROH>8**_	***F***_**ROH>16**_
**Brown Swiss**	**50 k panel**	**A**	0.154^b^	0.129^ab^	0.103^a^	0.073^ab^	0.039^dfhi^
		**B**	.	.	.	.	0.036^bceg^
	**HD panel**	**C**	0.151^b^	0.132^b^	0.109^b^	0.079^c^	0.042^i^
		**D**	0.147^a^	0.128^a^	0.105^a^	0.076^b^	0.040^gh^
		**E**	.	0.129^ab^	0.105^a^	0.075^b^	0.038^ef^
		**F**	.	.	0.105^a^	0.071^a^	0.035^cd^
		**G**	.	.	.	0.068^a^	0.033^b^
		**H**	.	.	.	.	0.028^a^
**Pinzgauer**	**50 k panel**	**A**	0.069^c^	0.048^ab^	0.037^a^	0.026^bc^	0.014^f^
		**B**	.	.	.	.	0.013^ed^
	**HD panel**	**C**	0.062^b^	0.049^b^	0.039^b^	0.027^d^	0.014^fe^
		**D**	0.060^a^	0.048^ab^	0.038^ab^	0.026^dc^	0.013^fe^
		**E**	.	0.048^a^	0.037^a^	0.026^bc^	0.012^d^
		**F**	.	.	0.036^a^	0.025^ab^	0.012^c^
		**G**	.	.	.	0.024^a^	0.011^b^
		**H**	.	.	.	.	0.008^a^
**Tyrol Grey**	**50 k panel**	**A**	0.080^c^	0.054^a^	0.042^a^	0.029^abc^	0.017^df^
		**B**	.	.	.	.	0.015^ce^
	**HD panel**	**C**	0.066^b^	0.052^a^	0.042^a^	0.030^c^	0.017^f^
		**D**	0.063^a^	0.051^a^	0.041^a^	0.029^b^	0.016^d^
		**E**	.	0.051^a^	0.040^a^	0.029^b^	0.016^ed^
		**F**	.	.	0.040^a^	0.028^ab^	0.015^c^
		**G**	.	.	.	0.026^a^	0.013^b^
		**H**	.	.	.	.	0.010^a^

Definition of Class is according to the number of heterozygous SNPs allowed within ROH length categories (see Table 2).

*F*_ROH_ values were obtained by allowing different numbers of heterozygous SNPs in each ROH length category; different letters indicate statistical significance within the same column and breed (P < 0.05, paired *t*-test); P values were corrected for multiple tests using the step-down Bonferroni method of Holm [18].

**Figure 3:**
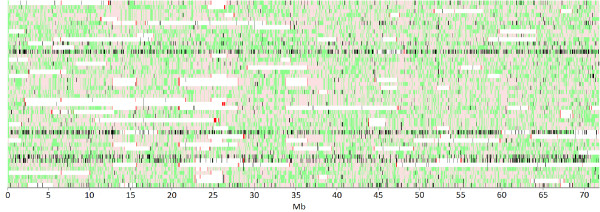
**Visualization of SNP data of chromosome 20 in Brown Swiss animals.** Light pink and light green colors represent homozygous and heterozygous SNPs, respectively; ROH are represented by white blocks, while missing SNPs are indicated in black; red lines within ROH indicate the presence of heterozygous SNPs; each row represents one animal.

At the same time, the number of allowable heterozygous calls should be limited. On the one hand, SNP data from chromosome 20 in the 46 Brown Swiss cattle (Figure 3) shows clearly that single, potentially miscalled heterozygous SNPs would interrupt ROH segments if such SNPs were not allowed. On the other hand, the figure also shows that allowing certain minimum numbers of heterozygous SNPs leads to inaccurate ROH calling at the ends of ROH. Such inaccurate calling is also likely to be a problem in individual ROH, since we sometimes observed multiple heterozygous SNPs close together within a ROH, not only when using the SNP & Variation software suite but also when using other programs (PLINK; [16]; cgaTOH; [22]; data not shown). In any event, ROH identification software should be improved so that instances of multiple heterozygous SNPs very close to one another should automatically lead the program to define separate ROH. Until such an improvement is made, we recommend careful visual analysis of ROH segment structure in order to exclude spurious ROH.

### Inbreeding coefficients estimated from ROH and ROH distribution

The HD panel gave the following mean *F*_ROH_ values across all ROH lengths: Brown Swiss, 0.151; Pinzgauer, 0.062; and Tyrol Grey, 0.066. Short ROH, i.e. 1 to 2 Mb long, covered an average of 36.7 Mb of the 2.3 Gb of the autosomal cattle genome covered with SNPs (Figure 2), with the highest short-ROH coverage observed in Brown Swiss and the lowest in Pinzgauer, the total genome length covered by all ROH > 1 Mb was 24.5% for one Brown Swiss bull and 23.0% for one Tyrol Grey bull. ROH > 16 Mb covered an average of 66.1 Mb of genome, although this number varied widely from animal to animal and between breeds. The highest long ROH coverage was observed in Brown Swiss and the lowest in Pinzgauer cattle. Some animals lacked such long ROH, whereas others showed a few that covered more than 300 Mb. The greatest genome coverage by long ROH was observed in a Tyrol Grey bull, in which 12 long ROH segments covered 368.6 Mb, corresponding to an average segment length of ≈30 Mb. The length of an autozygous segment indicates its age; since haplotypes are broken up by meiotic recombination, a short autozygous region is likely to have an ancient origin, while a long one probably arose recently [2,4]. These findings suggest that the Brown Swiss breed experienced both recent and ancient inbreeding events to a higher degree than the two other breeds.

Correlations of *F*_ROH_ values across all ROH lengths with pedigree inbreeding coefficients were similar to those previously reported by Ferenčaković et al. [8]. Correlations for the 50 k panel were 0.62, 0.65 and 0.77 for Brown Swiss, Pinzgauer and Tyrol Grey, respectively, and corresponding values were 0.61, 0.62 and 0.75 for the HD panel. Differences in correlations between panels within breeds were not statistically significant. Variation of these values is most likely due to the fact that pedigree-based inbreeding coefficients do not account for variation in meiosis, inheritance of segments of chromosomes and LD.

**Figure 4:**
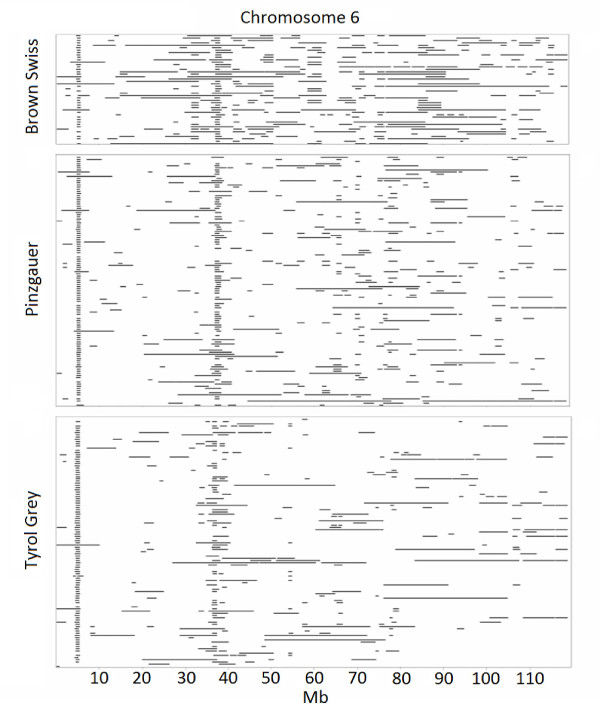
**ROH patterns on chromosome 6.** ROH on chromosome 6 from Brown Swiss, Pinzgauer and Tyrol Grey bulls identified using HD panel data; each row represents one animal.

The genomic distribution of ROH based on the HD panel data shows that 99.98% of SNPs occurred within an ROH of at least one individual. However, the frequency with which different SNPs occurred within ROH was not uniform across the genome, revealing genomic regions with abundant ROH, called ROH hotspots, which are also often detected in human populations [23,24]. Several ROH hotspots were common to all three breeds. For example, two hotspots were identified on chromosome 6 in all three breeds: one at 5.3-6.3 Mb and another at 38.4-39.5 Mb (Figure 4). Why these hotspots occur, and how they compare among cattle breeds and with other animal species, are questions currently under investigation.

## Conclusions

ROH identification in cattle is usually performed with the Illumina BovineSNP50 Genotyping BeadChip (50 k panel) or the Illumina BovineHD Genotyping BeadChip (HD panel). Here, we report that data from the 50 k panel do not represent the true state of autozygosity well for short ROH segments, while it is as reliable as the HD panel data for ROH > 4 Mb. When shorter segments are included with the 50 k panel, *F*_ROH_ is systematically overestimated. The bias due to potential genotyping errors depends on the allowance of heterozygous genotypes in a ROH calling software. While not allowing for heterozygous calls often just splits a very long ROH in two shorter ones that are still recognized and therefore the level of autozygosity of an individual is virtually unaffected, there are many cases where the shorter part of the split does not reach the minimum size of a ROH and the level of autozygosity of an individual is underestimated. Allowing many heterozygous calls in an ROH adds many short segments that are most likely not autozygous to the terminal regions of ROH. Our aim was to provide guidelines to identify ROH from high-throughput SNP genotype data. First, quality control should be performed by removing SNPs based on strict limits on genotype quality scores provided to reduce genotyping errors. Second, the number of heterozygous SNPs allowed should be determined separately for each ROH length of interest and for each SNP density, as suggested here. Third, if multiple heterozygous SNPs are allowed within the same ROH, adjacent heterozygous SNPs should be treated differently from heterozygous SNPs that are further apart. Because no current ROH identification software takes care of adjacent heterozygous SNPs, careful visual inspection of ROH segments should be applied to exclude spurious ROH called by the software.

## Competing interests

The authors declare that they have no competing interests.

## Authors’ contributions

MF participated in the design of the study, identified ROH, performed all data and statistical analyses, and drafted the manuscript. JS and IC conceived the study, helped to prepare figures and tables, and revised the manuscript. All authors read and approved the final manuscript.
